# Pathways to Next‐Generation Fire‐Safe Alkali‐Ion Batteries

**DOI:** 10.1002/advs.202301056

**Published:** 2023-06-19

**Authors:** Yubai Zhang, Jiabing Feng, Jiadong Qin, Yu Lin Zhong, Shanqing Zhang, Hao Wang, John Bell, Zaiping Guo, Pingan Song

**Affiliations:** ^1^ Centre for Future Materials University of Southern Queensland Springfield 4300 QLD Australia; ^2^ Queensland Micro Nanotechnology Centre School of Environment and Science Griffith University Nathan Campus 4111 QLD Australia; ^3^ Centre for Catalysis and Clean Energy School of Environment and Science Griffith University Gold Coast Campus 4222 QLD Australia; ^4^ School of Chemical Engineering & Advanced Materials The University of Adelaide Adelaide 5005 SA Australia; ^5^ School of Agriculture and Environmental Science University of Southern Queensland Springfield 4300 QLD Australia

**Keywords:** alkali‐ion batteries, battery materials design, fire safety, safety evaluation, thermal management

## Abstract

High energy and power density alkali‐ion (i.e., Li^+^, Na^+^, and K^+^) batteries (AIBs), especially lithium‐ion batteries (LIBs), are being ubiquitously used for both large‐ and small‐scale energy storage, and powering electric vehicles and electronics. However, the increasing LIB‐triggered fires due to thermal runaways have continued to cause significant injuries and casualties as well as enormous economic losses. For this reason, to date, great efforts have been made to create reliable fire‐safe AIBs through advanced materials design, thermal management, and fire safety characterization. In this review, the recent progress is highlighted in the battery design for better thermal stability and electrochemical performance, and state‐of‐the‐art fire safety evaluation methods. The key challenges are also presented associated with the existing materials design, thermal management, and fire safety evaluation of AIBs. Future research opportunities are also proposed for the creation of next‐generation fire‐safe batteries to ensure their reliability in practical applications.

## Graphical abstract

A multidisciplinary yet critical review of the development of fire‐safe alkali‐ion batteries is given, by analyzing the cutting‐edge materials design strategies and discussing the current thermal management methods as well as the battery safety evaluation approaches. It is highly advocated to integrate the study of battery thermal runaway characterization and fire safety evaluation into the high‐performance battery materials design.

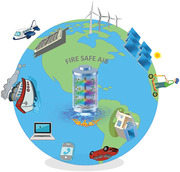

## 1 Introduction

Energy storage devices have been intensively studied for renewable energy storage in electricity grids and for powering electric vehicles and portable electronics.^[^ ^1^ ^]^ Of all the rechargeable energy storage devices, LIBs have already become the most widespread choice because of their high energy density, low self‐discharge, high output voltage, no memory effect, and low cost of maintenance.^[^ ^2^ ^]^ Meanwhile, despite their massive industrial applications, the dearth and uneven global distribution of lithium have catalyzed the exploration of other inexpensive and more sustainable AIBs. For instance, sodium‐ and potassium‐ion batteries (denoted as SIBs and PIBs, respectively) are also intensively studied, sharing the rocking‐chair energy storage mechanism with LIBs.^[^ ^3^ ^]^

**Figure 1:**
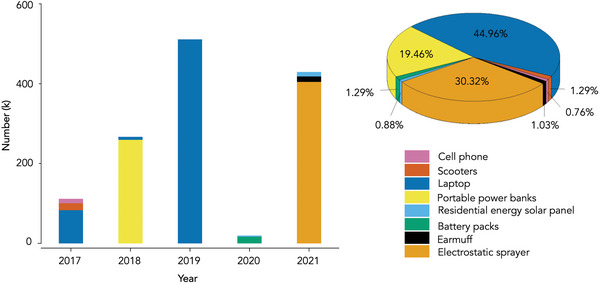
The number of LIBs–related electric consumer products recalled in the past 5 years. Relevant data is based on the search result from the official website of the United States consumer product safety commission.

Unfortunately, LIBs continue to be beset by their inherent fire issues, especially with their accelerated applications in a variety of electronics and electric vehicles.^[^ ^4^ ^]^ Battery fires (sometimes, even explosions) are repeatedly reported due to the battery failure that leads to thermal runaway in service.^[^ ^4^, ^5^ ^]^ For this reason, great efforts have been made to improve the fire safety of LIBs and to ensure the accident frequency rate is less than one per million LIB cells, but the results remain far from satisfactory. This issue is particularly critical for grid‐scale applications, given that at least hundreds of LIB cells are connected in series in a single battery pack which can exponentially increase the fire risk.^[^ ^6^ ^]^ Recently, there have been some severe fires caused by failed LIBs. Along with the battery fires, the last five years have also witnessed a large number of product recalls directly due to the use of defective, unsafe LIBs (see Figure 1). Therefore, it is critical to create fire‐safe batteries for mitigating and avoiding their thermal runaway. This can be achieved through designing fire‐safe battery materials and improving the battery thermal management system.

In general, thermal runaway can occur in the Li/Na/K ion batteries via a similar mechanism that involves a series of key chemical reactions in three main stages.^[^ ^7^ ^]^ Specifically, the onset overheating of the battery is the initial stage of thermal runaway, which can happen under normal charging/discharging processes and also abnormal conditions.^[^ ^8^ ^]^ With an increase in temperature, AIBs undergo exothermic chemical chain reactions.^[^ ^9^ ^]^ These reactions, in sequence, involve the decomposition of the solid electrolyte interphase (SEI) on the anode surface, the reaction of intercalated alkali ions in the anode with organic solvents from the electrolyte, the melting of the polyolefin‐based separators, and the decomposition of active cathode materials.^[^ ^7^, ^10^ ^]^ Finally, thermal runaway occurs when the combustion of flammable electrolytes with low flashpoints is triggered by the accumulated heat and oxygen gas released from these side reactions.^[^ ^8^, ^11^ ^]^

To enhance the fire safety of batteries, considerable efforts have been made in materials design, thermal management, and the incorporation of safety control devices over the past two decades.^[^ ^7c,f^ ^]^ The materials design mainly focuses on the use of more oxidation‐tolerant or fire‐safe battery components,^[^ ^8b^ ^]^ e.g., thermally stable cathode materials, non‐flammable liquid electrolytes and solid‐state electrolytes (SSEs). Additionally, the shut‐down separators, as an internal safety mechanism, have been reported to curb thermal runaway by rapidly decreasing the ionic conduction once the internal temperature of the battery cell exceeds a set limit, cutting off the cell charge/discharge.^[^ ^12^ ^]^ Thermal management units can be integrated into LIB cells, modules, and packs to protect against abnormal conditions.^[^ ^13^ ^]^ For example, cooling coating layers or air cooling systems are capable of dissipating excessive thermal energy generated by the battery packs in the automotive and aerospace industries.^[^ ^14^ ^]^ Safety devices are also employed to enhance the fire safety of batteries, and they include cell vents or tear‐away tabs, current interrupt devices (CID), positive temperature coefficient (PTC) disks, current‐limiting fuses, diodes, and battery management systems (BMS). Beyond the fire prevention strategies, a specific and systematic fire safety evaluation for AIBs is necessary to ensure their reliability in varied working environments.^[^ ^13^ ^]^

**Figure 2:**
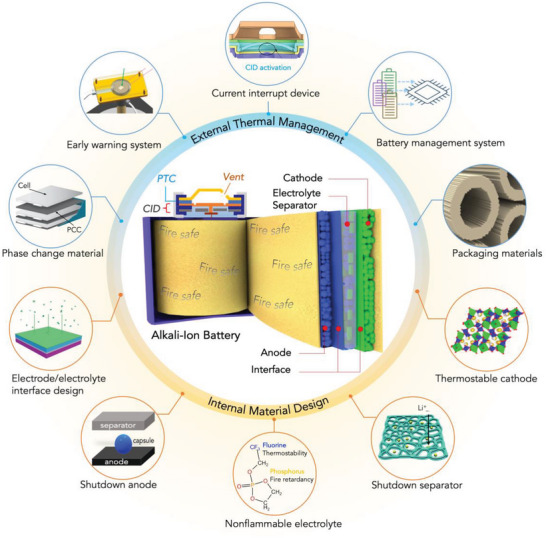
Schematic illustration for the creation of fire‐safe AIBs via internal materials design and external thermal management. External thermal management approaches include phase change materials (PCMs) for thermal management (Reproduced with permission,^[^ ^15^ ^]^ Copyright 2016, Wiley‐VCH,), the resistance temperature detector (RTD) (Reproduced with permission,^[^ ^16^ ^]^ Copyright 2019, Springer Nature), CID (Reproduced with permission,^[^ ^17^ ^]^ Copyright 2020, Elsevier), BMS, and thermal insulation packaging materials (Reproduced with permission,^[^ ^18^ ^]^ Copyright 2018, Springer Nature). Internal materials design is consisting of solid electrolyte for stable SEI of LIB (Reproduced with permission,^[^ ^19^ ^]^ Copyright 2022, Wiley‐VCH), thermally induced shut‐down anode (Reproduced with permission,^[^ ^20^ ^]^ Copyright 2012, Wiley‐VCH), non‐flammable electrolytes (Reproduced with permission,^[^ ^21^ ^]^ Copyright 2020, Springer Nature), thermally induced shut‐down separator (Reproduced with permission,^[^ ^22^ ^]^ Copyright 2017, The Royal Society of Chemistry), and the thermally stable cathode (Reproduced with permission,^[^ ^23^ ^]^ Copyright 2013, American Chemical Society).

To date, the study of fire safety of the AIBs has been given a priority in energy storage. A holistic review is therefore important in guiding the development of next‐generation fire‐safe AIBs. In this review, we first provide valuable insights into the causes of battery fires and then review the recent advances in creating fire‐safe AIBs based on internal materials design and external thermal management, as summarized in **Figure** **2**. We also outline the core design principles for improving thermal safety of electrodes and electrolytes, as well as inhibiting internal short‐circuiting induced fire hazards by developing solid‐state electrolytes and multifunctional separators. Moreover, we discuss the existing fire safety evaluation methods for batteries, considering the thermal stability and flammability of battery materials, thermal runaway characteristics and fire hazards of batteries. Finally, we provide directions for future research on materials design, thermal management and the fire safety evaluation of batteries.

## 2 Origin and Mechanism for AIBs Fires

Commercial LIBs are extremely sensitive to high temperatures (>60 °C) which can trigger abnormally fast degradation of battery packs. The thermal risk can be exacerbated in the presence of flammable organic liquid electrolytes in LIBs, which can cause fast fire propagation from a single cell to the whole LIB pack, causing the package to catch fire. To achieve fire‐safe batteries, understanding the origin and mechanism of AIB fires is the key to the battery materials design and the smart BMS, especially the battery thermal management system (BTMS).

The fire of LIBs is generally triggered by the exothermic reactions, often due to short‐circuits, exposing to high temperatures, and overcharging of batteries. The exothermic reaction could then result in a self‐enhanced increasing temperature loop named “thermal runaway” leading to battery fires or even explosions. The onset of thermal runaway is typically defined as the temperature at which the rate of heat generation exceeds the rate of heat dissipation, leading to a self‐sustaining increase in temperature.^[^ ^24^ ^]^ A widely accepted criterion for identifying the onset of thermal runaway is when the battery temperature rate surpasses 1 °C s^−1^.^[^ ^25^ ^]^ AIBs are particularly prone to thermal runaway under three kinds of extreme conditions, including mechanical stress (such as crushing or penetration), electrical factors (such as overcharging, internal and external short circuits), and thermal stress (such as overhearing). The mechanical abuse which may tear the separator in the battery, can result in the contact of cathode and anode and cause internal short circuit. Electrical abuse is generally related to the failure of BMS, leading to abnormal operation of batteries, such as overcharging, resulting in dendrites growth and internal short circuit. Thermal abuse, or overheating will trigger unwanted chemical reactions inside batteries, such as decomposition of SEI under high temperatures.^[^ ^26^ ^]^ By integrating fire‐retardant materials into the battery design, it can either self‐extinguish or shut down when subjected to abuse conditions that can result in degraded battery performance or thermal runaway.

**Figure 3:**
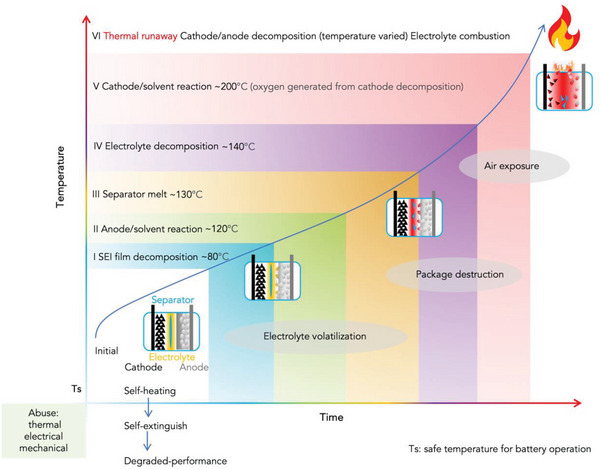
Schematic of the classification of thermal runaway main stages of AIBs. Reproduced with permission,^[^ ^27^ ^]^ Copyright 2022, American Chemical Society.

Study has shown the initial decomposition of SEI membrane was observed at the very beginning of thermal runaway, at temperature about 80 °C (see **Figure** **3**).^[^ ^28^ ^]^ The exothermal decomposition of SEI will generate O_2_, CO_2_ and C_2_H_4_ gaseous. Followed with the decomposition of SEI, the alkali intercalated anodes are directly exposed to the electrolytes, and the intercalated alkali metal will react with electrolytes at around 120 °C.^[^ ^29^ ^]^ Once the temperature increases to the melting point of commercial polyolefin separator (≈130 °C), the shrinkage and collapse of separator will lead to internal short circuit.^[^ ^25^ ^]^ The decomposition of liquid solvent in electrolytes will start at 122 °C for diethyl carbonate (DEC) and 140 °C for ethylene carbonate (EC) in inert atmosphere,^[^ ^30^ ^]^ which may generate gaseous CO and C_2_H_4_ that destruct the battery package.^[^ ^31^ ^]^ At the temperature of ≈140–150 °C, the cathode materials, such as Ni‐rich layered oxide and LiCoO_2_, will start decomposing and generating O_2_.^[^ ^32^ ^]^ The O_2_ will react aggressively with carbonate electrolyte at the cathode/solvent interface with huge heat generation accelerating the self‐heating rate of battery.^[^ ^33^ ^]^ When compared to Ni‐rich layered oxide and LiCoO_2_, LiFePO_4_ demonstrates greater thermal stability and does not release O_2_. While overcharged LiFePO_4_ lithium‐ion cells experience thermal runaway earlier than Ni‐rich oxide cells, indicating their lower overcharge tolerance, in the event of thermal runaway, LiFePO_4_ cells only release a significant amount of smoke without igniting fire.^[^ ^34^ ^]^ Finally, the rapid rise in the battery temperature becomes out of control, leading to electrode decomposition and electrolyte combustion.^[^ ^35^ ^]^ A fire in an individual cell can propagate to neighboring cells and, as a consequence, ignite the whole battery module or pack (Figure 3).

Studies on battery thermal runaway suggest that the higher the energy density batteries are more prone to catastrophic fires and explosions. Therefore, understanding the detailed thermal runaway characteristics of high energy AIBs, materials failure behavior and mechanisms, and thermal runaway mitigation technologies is crucial for directing fire safety research on batteries. Wang et al. reviewed the challenges of designing intrinsically fire‐safe high‐energy LIBs, emphasizing the importance of studying the thermal runaway characteristics of the whole battery system, particularly the chemical crosstalk inside cells during thermal runaway.^[^ ^36^ ^]^ Recent research has focused on developing fire‐safe battery components, proponing the onset temperature of thermal runaway, and reducing the heat release amount.^[^ ^37^ ^]^ The thermal behaviors of AIBs components under thermal runaway differ due to their distinct chemical properties.^[^ ^38^ ^]^ In Section 3, we will comprehensively review and compare the latest investigated AIB materials’ thermal behavior and design strategies to guide next‐generation fire‐safe AIB design.

## 3 Battery Materials Design

Thermal runaway is likely to occur as the battery temperature goes beyond a critical point that the battery materials cannot withstand. Therefore, the design of fire‐safe materials or AIBs needs to start by addressing the thermal stability of cell components, including electrodes, electrolytes, separators, and the whole battery system.^[^ ^26a^ ^]^

### 3.1 Anode Design

#### 3.1.1 LIBs Anodes

In the past two decades, graphite and silicon materials have been used as negative electrode materials because they are safer and more thermostable alternatives to metallic Li anodes.^[^ ^39^ ^]^ However, they are also not immune to Li dendrite growth which could cause internal short‐circuits, especially at either high current densities or under overcharging conditions.^[^ ^40^ ^]^ Homogenizing the Li‐ion flux during Li deposition by applying protective layer coating on the anode and artificial SEI engineering has been reported to be a promising solution for mitigating the dendrite growth.^[^ ^26^, ^41^ ^]^ Moreover, ensuring higher chemical and thermal stability in the artificial SEI is crucial to delay the onset temperature of thermal runaway. The SEI of graphite anode in conventional electrolyte containing LiPF_6_ in EC and EMC typically comprises lithium oxalate (Li_2_C_2_O_4_), lithium carboxylate (RCOOLi), lithium methoxide (LiOCH_3_), LiOH, and LiHCO_3_.^[^ ^42^ ^]^ However, residual products formed during battery cycling, such as PF_5_ and H^+^ in LiPF_6_‐based electrolytes, can attack SEI.^[^ ^43^ ^]^ To address this issue, researchers have used LiFSI to replace LiPF_6_ in electrolytes or added Lewis‐basic tris(2,2,2‐trifluoroethyl) phosphite (TTFP) in electrolytes to prevent SEI damage. LiFSI is particularly useful as it generates no Lewis acidic species within the high temperature range of ≈60–90 °C, thus avoiding SEI damage caused by acidic species. TTFP can scavenge Lewis‐acidic PF_5_ by forming an acid‐base adduct and promote the formation of thermally stable SEI by forming LiF and CF_3_CH_2_O‐ groups that enhance thermal stability.^[^ ^44^ ^]^ Lewis‐basic zeolites have also been reported to trap H^+^ species generated during battery operation, preventing SEI chemical degradation.^[^ ^45^ ^]^ Inorganic components, such as LiF, Li_2_O, can also be incorporated into SEI to form low‐resistance, thermally stable SEI that perform well under thermal abuse.^[^ ^46^ ^]^ Lithium bis(oxalate)borate (LiBOB) can form a thick, thermally stable SEI upon reduction in the cell, postponing the onset temperature of SEI decomposition to 150 °C.^[^ ^47^ ^]^ More recently, Wu et al. reported using 1,3,5‐trimethyl‐1,3,5‐tris(3,3,3‐trifluoropropyl)cyclotrisiloxane (D_3_F) in electrolytes to passivate the lithiated graphite anode. Targeted repair of SEI by D_3_F can inhibit exothermic reactions in the early stage of thermal runaway for LIBs.^[^ ^48^ ^]^ In addition, high‐concentration electrolytes (>3M) and localized high‐concentration electrolytes can facilitate the formation of inorganic‐rich SEI, leading to enhanced interfacial stability of batteries due to their unique solvent structures.^[^ ^49^ ^]^ As SEI characterization advances, such as through nano‐scale in situ and operando characterization methods, SEI manipulation can be effectively used to enhance the thermal stability of LIB anodes.

Another safety issue for these anode materials arises from the exothermic reactions of intercalated or alloyed Li in graphite or silicon with the electrolytes in the absence of the SEI layers, which means that more heat is released at a higher lithiation level.^[^ ^41a^ ^]^ One facile solution to this issue is the introduction of thermal‐responsive polymers into anodes. For instance, poly(ethylene) (PE) can be incorporated into the anodes as a thermal runaway firewall, which can cut off the electric connection between the cell components by either foaming or melting under higher temperatures (≈95–110 °C).^[^ ^20^, ^50^ ^]^ Besides, flame retardant additives in the anodes can efficiently prevent the ignition of materials, for example, flame retardant binder, polyacrylic acid (PAA) cross‐linked with phosphorus and nitrogen‐containing epoxy resin, can be incorporated to form a fire‐safe Si anode without sacrificing the battery performance.^[^ ^51^ ^]^

Above all, the thermal risk of LIB anode mainly comes from the intercalated Li with liquid electrolyte, strategies including adding fire‐retarding binder in anodes, and creating SEI with enhanced thermal and chemical stability shed lights on increasing LIB's thermal safety. Furthermore, the utilization of a high thermally conductive anode can aid in the dissipation of heat for LIBs. Graphene has been demonstrated to be an effective thermal conductive agent in the anode, as it can significantly enhance the thermal conductivity of SnO_2_‐based anodes up to 535.3 W m^−1^ K^−1^.^[^ ^52^ ^]^

#### 3.1.2 SIBs Anodes

For next‐generation SIBs, cheap hard carbon materials are commonly adopted for anodes rather than graphite, because Na^+^ ion intercalated graphite is thermodynamically unstable presenting a low capacity of 35 mAh g^−1^.^[^ ^53^ ^]^ However, the low working potential plateau (<0.2 V) of hard carbon can lead to safety issue due to dendrite formation, and degraded long cyclic performance.^[^ ^54^ ^]^ Similar to graphite and silicon anodes of LIBs, the interspatial stability of hard carbon with electrolytes is known to be playing a key role in hindering Na dendrites formation as well as battery cyclic stability. Studies show that fluoroethylene carbonate (FEC) additives in electrolyte could assist in forming stable SEI. Besides, by shortening the Na^+^ transport path in microstructured hard carbon, the working potential of hard carbon could be increased to avoid dendrites formation.^[^ ^55^ ^]^ These two strategies are considered to be effective in improving hard carbon anodes’ safety in SIBs.

Besides hard carbon, of which the capacity is around 330 mAh g^−1^, novel high capacity anode materials for SIBs are being explored in recent years. Zhou et al. reported nanosized red phosphorus and reduced graphite oxide composite anode, the former of which not only delivers high energy density (theoretical capacity of 2596 mAh g^−1^) but also imparts flame retardancy to the composite anode.^[^ ^56^ ^]^ During the combustion of electrolytes and anodes, a phosphoric acid derivative will form in the composite anode, and it can isolate the burning material from oxygen and catalyze char layer formation on the material surface, thus prevent flame formation. Besides, other alloy‐ and compound formation‐type anodes (e.g., Sn, Si, Sb) are also investigated as SIB anodes, but often show low cyclic stability due to the significant volume changes during de/sodiation process, resulting in particle pulverization, loss of electrical contact, and unstable SEI. To tackle this issue, structural design similar to core‐shell structured Si anodes for LIBs could be applied, but the complicated fabrication process will increase the cost.^[^ ^57^ ^]^

In another aspect, the exothermic reaction of the sodiated alloy and/or compound with electrolytes will cause safety concern. For instance, Lee and coworkers discovered that the exothermic heat generation of the reaction between the sodiated Sn anode and the electrolyte in SIB is significantly higher than the Sn anode in LIB.^[^ ^58^ ^]^ It highlights the importance of evaluating the thermal stability of the developed SIB anodes for practical application.

#### 3.1.3 PIBs Anodes

In terms of fire‐safe PIB anode, low‐cost graphite is one of the popular choices, because K^+^ ion can be intercalated into graphite to form a stable structure with a high capacity of 279 mAh g^−1^.^[^ ^59^ ^]^ From Adams et al.’s work about the thermal runaway elucidation of PIB with graphite anode, it is known that compared to the analogous LIB system, the K‐intercalated graphite anode evolves less heat (1048 vs 395 J g^−1^) during thermal runaway. From this aspect, graphite is relatively a safe anode for PIBs. The only drawback of graphite anode in PIB is the huge volume change during K^+^ ion de/intercalation due to its larger ionic size than Li^+^ ion, which can lead to capacity decay during cycling.

Alternatively, hard carbon is a good choice for PIB anode, which delivers relatively stable cycling stability. The thermal runaway of PIB using hard carbon anode remains unexplored to date, so more fundamental research is demanded. Like SIBs, metalloid‐based alloying anodes are applied for PIBs. Among them, layered phosphorous‐like GeP_5_ shows a comparable high and stable capacity of 213.7 mAh g^−1^ over 2000 cycles, and GeP_5_ is intrinsically high thermally stable.^[^ ^60^ ^]^ Wu et al. have reviewed the challenges and promises of phosphorus‐based anode for PIBs in detail.^[^ ^61^ ^]^ The development of high energy‐density phosphorus‐based anodes paves the way for fire‐safe and high‐performing PIBs.

#### 3.1.4 Alkali Metal Anodes

Despite the high reactivity of alkali metals, alkali metal batteries, in which alkali metal is used as anodes, are considered a “holy grail” for rechargeable batteries because of its ultrahigh theoretical capacity (e.g., 3860 mAh g^−1^ of Li metal).^[^ ^62^ ^]^ As they are susceptible to dendrites formation causing short circuits and thermal runaway, their practical applications are seriously hampered. There is a growing number of strategies being discovered to tackle the dendrites concern of Li metal anode. Bao et al. have provided a comprehensive review about taking advantage of the crystallographic optimization to suppress the dendrite growth.^[^ ^63^ ^]^ In addition, 2D materials have been combined with alkali metal to construct safer composite anodes.^[^ ^64^ ^]^ For instance, for PIBs, because of its low melting point (64 °C),^[^ ^65^ ^]^ the K metal anode needs to be embedded in a host matrix (e.g., reduced graphene oxide), and the composite anode could show a good ability to maintain the solid structure without the loss of K at 200 °C.^[^ ^66^ ^]^ Embedding fire‐retarding host matrix materials in alkali metal anodes can make provision for improving its non‐flammability and thermal stability.

### 3.2 Cathode Design

Regarding fire‐safe cathode design for AIBs, it mainly aims at averting the decomposition of active materials and the oxygen evolution at elevated temperatures or voltages. Surface coating and chemical composition modification are proven two promising approaches to effectively suppress oxygen release and mitigate the thermal runaway of batteries.^[^ ^67^ ^]^

#### 3.2.1 LIBs Cathodes

The cathode chemistry plays a definitive role in the thermal instability of batteries. Among the conventional cathode materials, LiFePO_4_ is considered to be the most thermally stable cathode, because the unstable layered oxides cathodes would generate oxygen upon heating.^[^ ^68^ ^]^ The commonly used LiCoO_2_ cathode tends to decompose above 180 °C along with a dramatic heat release, which can trigger thermal runaway.^[^ ^7f^ ^]^ To eliminate this exothermic reaction, Xia et al. coated the LiCoO_2_ particles with a thin layer of conductive polymer, poly(3‐decylthiophene), which has a positive temperature coefficient, thus can shut down the charge transfer in the cathode at 110 °C.^[^ ^69^ ^]^ Similarly, Cho et al. reported an AlPO_4_ coating to boost the thermal stability of LiCoO_2_.^[^ ^70^ ^]^ In contrast, LiFePO_4_ is considered a safer cathode material with higher thermal stability than LiCoO_2_.^[^ ^71^ ^]^ However, it has an energy density of 170 mAh g^−1^, which is lower compared to lithium Ni‐rich oxides that have an energy density of at least 180 mAh g^−1^).^[^ ^72^ ^]^

The high content of Ni in lithium Ni‐rich oxides can boost the energy density but the thermal stability is compromised. To improve its thermal stability, Sun and co‐workers reported a novel Li[Ni_0.64_Co_0.18_Mn_0.18_]O_2_ cathode material in which each particle comprises a Ni‐rich core surrounded by a concentration‐gradient outer layer.^[^ ^73^ ^]^ This material showed excellent thermal stability and was found to perform safely in the nail penetration test. Their work provides valuable guidance for designing highly thermally stable and high‐capacity intercalating cathode materials. Besides, the coating treatment (e.g., CaF_2_) and elemental doping (e.g., Nb) on commercial LiNi_0.8_Co_0.1_Mn_0.1_O_2_ (NCM811) and LiNi_0.85_Co_0.01_Mn_0.05_O_2_ (NCM85) can realize a promising improvement in battery electrochemical performance under high temperature by enhancing their structural stability during cycling.^[^ ^74^ ^]^ Recent research by Wang et al. highlighted the correlation between the battery cycling stability and the thermal stability of Ni‐rich cathode materials. They discovered that increasing the calcination temperature during the fabrication of Li[Ni_0.83_Co_0.12_Mn_0.05_]O_2_ (NCM83) materials will lead to improved thermal stability of materials, but damage the cyclability of the battery.^[^ ^75^ ^]^ Lithium Ni‐rich oxides possess a high commercialization potential but further structural and thermal stability optimization are needed.

#### 3.2.2 SIBs and PIBs Cathodes

SIBs and PIBs share the typical cathode materials, including polyanions, transition metal oxides and hexacyanoferrates,^[^ ^76^ ^]^ whereas hexacyanoferrates (Prussian blue and its analogs) are less thermally stable due to the exothermic interaction of their cyanide groups with electrolytes when the temperature reaches above 200 °C.^[^ ^77^ ^]^ In comparison, polyanionic compounds show superior thermal stability owing to the strong covalent bonding of oxygen atoms.^[^ ^76^, ^78^ ^]^ Additionally, their open structures can support the fast cationic diffusion and provide sufficient space to host large Na^+^ and K^+^ ions, thereby improving battery safety as well. Barpanda et al. found that Na_2_FeP_2_O_7_ polyanionic compound for SIBs delivered outstanding thermal stability without decomposition or oxygen evolution through heating to 600 °C.^[^ ^23^ ^]^

Considering the capacity of materials, transition metal oxides based on layered structures are more attractive for SIBs and PIBs than polyanions.^[^ ^79^ ^]^ However, similar to LIBs, the Na or K‐rich transition metal oxides also suffer from the oxygen release during cycling. Hence, improving their structural stability during cycling is being investigated continuously. Bruce et al. detailly studied the triggering factors in oxygen loss in the oxygen redox transition metal oxides (P2‐ Na_x_[A_y_Mn_1−y_]O_2_ (where A = Li or Mg)). It is found that the depletion of alkali ions in materials leaves the oxidized O underbonded causing O loss, and Na_0.67_Mg_0.28_Mn_0.72_O_2_ overcomes Na_0.78_Li_0.25_Mn_0.75_O_2_ with the higher stability of O in the lattice during Na^+^ and/or Li^+^ de/intercalation because of the Mn^4+^ and Mg^2+^ coordinating bonding with O.^[^ ^80^ ^]^ This work put forward a facile strategy for improving the O stability in transition metal oxide materials for both SIBs and PIBs. In addition to this, elemental substitution of Fe on Na_2/3_Ni_1/3_Mn_2/3_O_2_ can prevent the oxygen release under high voltage by Fe coordinating bonding with O.^[^ ^81^ ^]^ Besides the above mentioned Na‐rich transition metal oxides, among 38 types of Na‐rich Na_2_MO_3_ compounds (M represents transition metals), 4d transition metal‐based ones (Na_2_RhO_3_ and Na_2_PdO_3_) are especially expected to exhibit both high energy density and high structural stability according to density functional theory calculation.^[^ ^82^ ^]^ For PIBs, different from LiCrO_2_ and NaCrO_2_ which release lattice oxygen during irreversible phase transformation at high voltage, KCrO_2_ shows no oxygen release during the first K extraction cycle.^[^ ^83^ ^]^

### 3.3 Electrolyte Design

Thermal runaway in AIBs, usually caused by internal short‐circuits (often related to metal dendrite) and/or extreme external factors, can lead to the ignition of conventional flammable liquid electrolytes. This is due to the fact that such electrolytes act as fuel for fires in battery cells. To address this issue, a major step toward making batteries safer is to replace flammable electrolytes with non‐flammable alternatives.^[^ ^84^ ^]^ This review comprehensively covers non‐flammable electrolytes, including liquid electrolytes and SSEs, for LIBs, SIBs, and KIBs, based on reported reviews and the latest experimental findings.^[^ ^85^ ^]^

#### 3.3.1 Liquid Electrolytes

##### LIBs Liquid Electrolytes

Non‐flammable or flame‐retarding solvents, co‐solvents or additives,^[^ ^86^ ^]^ are being investigated as a means to enhance the nonflammability of liquid organic electrolytes.^[^ ^87^ ^]^ These compounds work by decomposing to create phosphorus or fluorine‐free radicals, which can scavenge active hydrogen and/or hydroxide free radicals that may be generated during thermal runaway of a battery.^[^ ^21^, ^84^, ^88^ ^]^ Non‐flammable solvents, such as alkyl phosphates, phosphazenes, and fluorinated carbonates, as well as fluorinated phosphazenes, are being explored for use in the organic electrolytes of LIBs.^[^ ^89^ ^]^ Studies have shown that phosphates, such as trimethyl phosphate (TMP), triethyl phosphate (TEP), and tripropyl phosphate (TPrP), can impart non‐flammability to electrolytes. However, high amounts of TEP and TMP (about 40 vol%) were required to achieve non‐flammability, and they are not compatible with graphite anodes, resulting in inferior cycling.^[^ ^90^ ^]^ Interestingly, the TEP‐based electrolytes can be compatible with lithium metal anodes, leading to high‐safety lithium metal batteries. Recently, Liao et al. reported that non‐flammable lithium salt, LiNO_3_, as the sole lithium salt in TEP and FEC organic electrolyte could significantly enhance the stable cycling and fire‐safety of lithium metal batteries.^[^ ^91^ ^]^ Guo et al. proposed a dual‐salt non‐flammable electrolyte, in which NO_3_^−^ anion is involved in the solvation structure of Li^+^ at a dilute state. In such an electrolyte, solvent molecules have a lower polarization degree and therefore less likely to decompose, achieving a plating/stripping efficiency of 99.49%.^[^ ^92^ ^]^

According to research, fluorides and fluorinated phosphazenes are more effective flame‐retardants and exhibit greater electrochemical stability compared to phosphates.^[^ ^89^ ^]^ A good example is a liquid electrolyte reported by Pham et al. in 2018 for full cell LIBs with graphite anode and Li_1.13_Mn_0.463_Ni_0.203_Co_0.203_O_2_ cathode. This electrolyte comprised 1 M Li salt (LiPF_6_) in propylene carbonate (PC), fluorinated DEC co‐solvent, and FEC additive is non‐flammable, and the assembled full cell with this electrolyte realized a stable charge–discharge cyclic stability, in contrast to quick capacity fade of full cell with conventional EC and ethyl methyl carbonate (EMC) electrolyte.^[^ ^93^ ^]^ An et al. also reported that 2,2,2‐trifluoroethyl acetate (TFA) can be added to PC based electrolyte to achieve fire resistance, and this TFA and PC‐based electrolyte incorporated full cell (graphite||LiNi_0.6_Co_0.2_Mn_0.2_O_2_) shows superior cycling stability than the EC and EMC based electrolyte battery system as well.^[^ ^94^ ^]^

**Figure 4:**
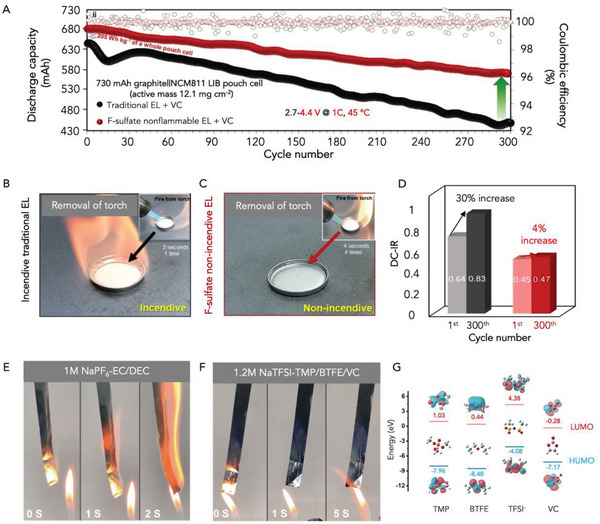
Fire‐retarding electrolytes designed for LIBs and SIBs. A) A non‐incendive liquid electrolyte containing fluorinated linear sulfate was used in LIB and achieved a high battery performance and fire safety compared to that traditional electrolyte. Flammability test results of B) traditional electrolytes and C) F‐sulfate non‐incendive electrolytes. D) Changes in direct current resistance of the pouch full‐cells before and after 300 cycles. Reproduced with permission,^[^ ^95^ ^]^ Copyright 2021, European Chemical Societies Publishing. Flammability tests for E) the conventional 1 M NaPF_6_‐EC/DEC and F) the 1.2 M NaTFSI‐TMP:BTFE:VC, and G) molecular orbital analysis on TMP, BTFE, TFSI^−^, and VC by DFT calculations. Reproduced with permission,^[^ ^107^ ^]^ Copyright 2021, Wiley‐VCH.

In 2021, Kwak and co‐workers used non‐flammable co‐solvents, fluorinated linear sulfate (bis(2,2,2‐trifluoroehtyl sulfate (FES)) and fluorinated ethyl methyl carbonate (FEMC), to prepare a fire‐safe organic electrolyte for full‐cell LIBs (graphite||NCM811).^[^ ^95^ ^]^ The addition of FES as a co‐solvent enhanced the thermal and anodic stability of the electrolytes and helped to form a robust and high Li^+^ ion conducting SEI, resulting in a superior cycling stability and non‐flammability in comparison to the conventional electrolyte (EC/EMC) (see **Figure** **4**A–C). By measuring the direct current‐internal resistance before and after cycling (see Figure 4D), this non‐flammable electrolyte showed a smaller resistance at the initial state, and only a marginal increase was observed after harsh cycling at 1 C and 45 °C for 300 cycles. While fluorine‐containing co‐solvents or additives show promising electrochemical behavior and fire safety in batteries,^[^ ^87^ ^]^ they are not environmentally friendly and are of high toxicity and cost. To address this issue, researchers have investigated EC‐free electrolytes such as triple‐salt EMC‐based electrolytes,^[^ ^96^ ^]^ and localized high concentration electrolytes,^[^ ^97^ ^]^ for LIBs and lithium metal batteries, respectively. In 2022, Wu et al. characterized the thermal safety of non‐flammable localized high‐concentration electrolytes for NMC811||graphite‐SiO pouch cells, which showed a significant increase in the thermal runaway triggering temperature.^[^ ^37^ ^]^

Despite the use of fire‐retarding electrolytes batteries can still pose a fire hazard. In 2021, Hou et al. characterized the thermal runaway process of LIBs employing flame‐retardant fluorinated electrolytes and found that the battery cannot self‐extinguish during a thermal runaway test.^[^ ^98^ ^]^ Further understanding of the thermal runaway of batteries employing fire‐safe electrolytes will be crucial in optimizing electrolyte design to prevent exothermic reactions with other battery components.

Organosilicon compounds that contain carbon‐silicon bonds offer several desirable properties, including low flammability, high thermal and electrochemical stability, and low toxicity.^[^ ^99^ ^]^ Additionally, fluorination of these compounds can yield fluorosilanes with low viscosity, high dielectric constant, and greater oxidative potential than non‐fluorinated organosilicon compounds. These fluorosilane compounds have demonstrated potential as safe electrolyte solvents for high‐energy‐density and safe LIBs.^[^ ^100^ ^]^

Ionic liquids, which are non‐flammable and stable, are a promising alternative for enhancing the fire‐safety of AIBs used in high‐temperature and high‐voltage applications.^[^ ^101^ ^]^ Compared to organic carbonates, the high coulombic attraction in ionic liquids requires more energy to break, making them less likely to explode at elevated temperatures.^[^ ^102^ ^]^ For example, while conventional polycarbonate electrolytes would explode at 150 °C, a pyrrolidinium‐based ionic liquid remains inert up to 300 °C.^[^ ^103^ ^]^ Additionally, by utilizing the catholically stable cation, such as ammonium, pyrrolidinium, and phosphonium, the electrochemical window of ionic liquid electrolytes can be expanded.^[^ ^104^ ^]^ However, the main challenge with using ionic liquid as electrolytes is their high cost and low ionic conductivity when compared to organic carbonates. To address this, optimizing the mixture of ionic liquids and organic electrolyte is a promising approach to improving ionic conductivity and reducing the amount of expensive ionic liquid used.^[^ ^105^ ^]^ A non‐flammable inorganic ionic liquid, LiAlCl_4_⋅3SO_2_, has been shown to deliver sufficient ionic conductivity of 23.77 mS cm^−1^ at room temperature for LIBs.^[^ ^106^ ^]^ These novel electrolytes offer high electrochemical performance and thermal safety, but full cell thermal runaway testing is still required.

##### SIBs and PIBs Liquid Electrolytes

For SIBs and PIBs, the use of conventional carbonate‐based electrolytes presents an even higher fire risk and falls short of forming a stable SEI layer on electrodes, owing to the higher reactivity of both Na and K than Li.^[^ ^88^, ^108^ ^]^ Similar to fire‐safe LIBs, phosphorus‐ and fluorine‐containing compounds are also applied as flame‐retardant additives or non‐flammable solvents for both SIBs and PIBs.^[^ ^88^, ^109^ ^]^ Yamada's group demonstrated a simple yet fire‐extinguishing electrolyte using a high concentration of NaN(SO_2_F)_2_ (for SIBs) or LiN(SO_2_F)_2_ (for LIBs) (3.3 M for both) as a salt and trimethyl phosphate (TMP) as a sole fire retardant solvent.^[^ ^88f^ ^]^ Such a highly concentrated electrolyte is very effective in passivating the carbonaceous anodes and forming a robust SEI layer to prevent the anodes from decomposition.^[^ ^88^, ^109^, ^110^ ^]^ In 2022, Chou's group designed a non‐flammable phosphorus‐containing electrolyte (1.2 M NaTFSI‐TMP/BTFE/VC) for pouch‐cell SIBs where the commercial hard carbon anode was matched to Na_3_V_2_(PO_4_)_3_ or Prussian blue cathode (see Figure 4E,F, 1 M NaPF_6_‐EC/DEC is the traditional electrolyte for SIBs).^[^ ^107^ ^]^ The use of high‐concentration electrolytes in practical applications is challenging due to their high viscosity. To address this issue, electrochemically “inert” and poorly solvating fluorinated ether (BTFE) is often utilized to reduce viscosity and enhance ionic conductivity. However, the addition of BTFE can disrupt the solvation balance in electrolytes. To restore the damaged Na^+^ solvation structure, a polar additive, vinylene carbonate (VC), is added. This effectively mitigates the negative impact of BTFE on solvation and improves overall electrolyte performance. As discerned by density functional theory (DFT), VC has a lower LUMO level than other constituents in the electrolyte (see Figure 4G).^[^ ^107^ ^]^ This makes VC more susceptible to decomposition and forms a passivating film on both electrodes to alleviate the battery aging.^[^ ^111^ ^]^

##### Aqueous Electrolytes

**Figure 5:**
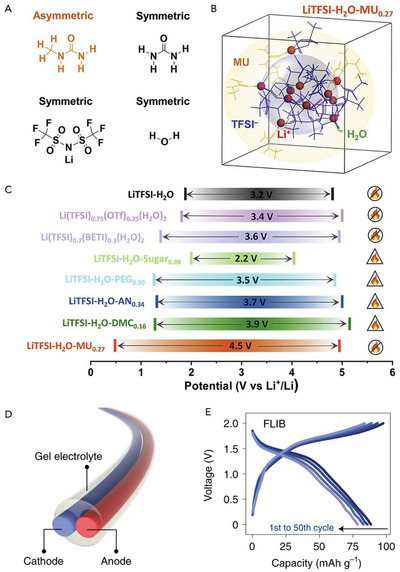
Design of non‐flammable aqueous/organic hybrid electrolytes for LIBs and industrial‐scale production of fiber LIB (FLIB) by solution‐extrusion approach. A) Asymmetric molecular structure of methylurea (MU) and B) the use of asymmetric donor‐acceptor MU to form a core‐shell‐like solvation structure in the aqueous electrolyte. C) The formulated LiTFSI‐H_2_O‐MU_0.27_ possesses a high 4.5 V window and a low hydrogen evolution reaction potential at 0.5 V versus Li^+^/Li compared to other aqueous electrolyte systems. Reproduced with permission,^[^ ^114^ ^]^ Copyright 2022, Elsevier. D) Schematic of fiber batteries fabricated by a solution‐extrusion method. Cathode and anode inks were fabricated by adding lithium manganese oxide and lithium titanate phosphate respectively in a slurry of acrylonitrile and styrene butadiene rubber binders with an aqueous dispersion of carbon nanotubes. Gel electrolyte inks were fabricated by adding polyvinyl alcohol and lithium sulfate (Li_2_SO_4_) to chitosan in acetic acid. E) The charge–discharge profiles of 10 cm‐long FLIB demonstrate that the fiber battery is fully functional with an average discharge potential stage of 1.3 V. Reproduced with permission,^[^ ^115^ ^]^ Copyright 2022, Springer Nature.

Relative to organic electrolytes, aqueous electrolytes are cheaper, greener, and intrinsically non‐flammable but the major bottleneck is their narrow electrochemical stability window (1.23 V) due to unwanted hydrogen evolution and electrode oxidation.^[^ ^112^ ^]^ To overcome this limitation, Wang and Xu reported the use of a water‐based electrolyte with an ultra‐high concentration of Li salt (LiTFSI, molality > 20 M) to broaden the electrochemical window to 3 V for aqueous LIBs.^[^ ^112b^ ^]^ The high salt concentration significantly alters the interfacial chemistry of the water‐in‐salt system. Quantum chemistry calculations indicate that the onset potential for LiTFSI reduction at this elevated concentration ranges from ≈2.7 to 2.9 V, slightly surpassing that of the hydrogen evolution process at 2.63 V. Consequently, by reducing the high concentration of TFSI prior to hydrogen evolution, a passivation process takes place, leading to the formation of a dense interphase on the anode surface. This effectively elevates the hydrogen evolution potential to 1.9 V, thereby enhancing the overall performance of the anode. On the cathode side, the increasing salt concentration appears to suppress the oxygen evolution, likely due to reduced water activity when coordinated to Li^+^ and an inner Helmholtz layer increasingly populated by TFSI anions. Following this work, they expanded the window to above 3.3 V by devising a ternary eutectic aqueous electrolyte (4.5 M LiTFSI–KOH–CO(NH_2_)_2_–H_2_O) which can promote a robust SEI layer.^[^ ^113^ ^]^ With this non‐flammable aqueous electrolyte, they fabricated a Li_1.5_Mn_2_O_4_||Li_4_Ti_5_O_12_ pouch cell showing good cycling life and a high energy density. In 2022, Lin et al. introduced non‐flammable methylurea (MU) to the aqueous electrolyte for eliminating the hydrogen evolution at 0.5 V versus Li^+^/Li, whereby a larger electrochemical window (4.5 V) was achieved.^[^ ^114^ ^]^ The asymmetric structured MU (**Figure** **5**A) can regulate the hydrogen bonding in the aqueous electrolyte by the coordination of MU to solvated Li^+^ and TFSI^−^ between carbonyl donor groups and amide acceptor groups. This also results in nanoscale core‐shell‐like clusters with localized ultra‐high Li salt concentrations (Figure 5B). This fire‐retardant water‐based electrolyte (LiTFSI‐H_2_O‐MU_0.27_) has a significantly lower cathodic limit and a wider electrochemical window than other aqueous electrolytes (as depicted in Figure 5C), thus contributing to supporting the stable operation of NbO_2_||LiMn_2_O_4_ full cell. Meanwhile, Peng and co‐workers reported a solution‐extrusion‐based fabrication method to fabricate continuous aqueous fiber LIBs and SIBs (Figure 5D,E).^[^ ^115^ ^]^ The incorporation of intrinsically fire‐safe aqueous gel electrolytes is crucial to improve the battery safety, especially for the target applications, i.e., compact wearable electronics. The quasi‐solid state aqueous electrolytes, which are non‐flammable, insensitive to air and water, and capable of preventing the leakage of aqueous solution, underpin their safe application in wearable devices.

Research on the thermal runaway of the AIBs with non‐flammable electrolytes is carried out to certify their realistic fire‐resistant performance. For instance, Hou et al. reported the LIBs with concentrated LiN(SO_2_F)_2_ in TMP electrolyte could still get triggered to thermal runaway with an onset temperature of 195.2 °C, similar to that of conventional electrolyte (213.1 °C), suggesting that the non‐flammability or even fire‐retardance of this liquid organic electrolyte cannot fulfill the fire‐resistance of the whole battery, the reaction between charged electrodes and electrolytes, as well as the exothermic reaction between contacting anode and cathode greatly contribute to thermal runaway.^[^ ^116^ ^]^ In the future research, the thermal runaway risks of non‐flammable liquid organic as well as aqueous electrolytes assembled AIBs should be scientifically evaluated by ARC.

#### 3.3.2 Solid‐State Electrolytes (SSEs)

SSEs are an appealing class of leak‐proof, separator‐free, and non‐volatile electrolytes with adequate electrochemical windows, and desired mechanical robustness to resist the dendrite penetration and volumetric change of electrodes during cycling.^[^ ^117^ ^]^ In general, three different types of SSEs, namely solid organic electrolytes (SOEs), solid inorganic electrolytes (SIEs), and solid composite electrolytes (SCEs), have been intensively investigated and hold great promise for fire‐safe AIBs applications.

**Figure 6:**
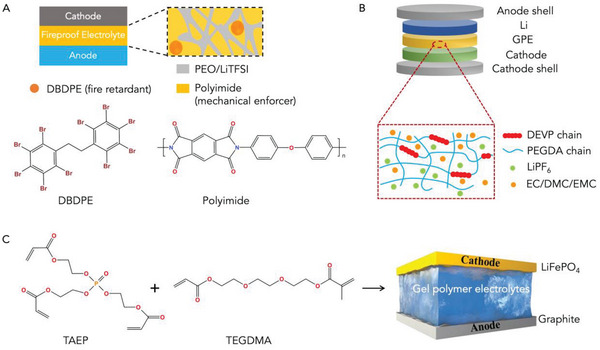
Schematic of compositions of fire‐safe SOEs. A) the PEO‐based (Reproduced with permission,^[^ ^120^ ^]^ Copyright 2020, American Chemical Society), B) PEG‐based SOEs (Reproduced with permission,^[^ ^123^ ^]^ Copyright 2022, Elsevier), C) In‐situ polymerization of TAEP and TEGDMA in liquid electrolyte for forming non‐flammable gel polymer electrolyte (Reproduced with permission,^[^ ^128^ ^]^ Copyright 2022, Wiley‐VCH).

Because of the less flammability than common organic liquid electrolytes, polyethylene glycol (PEG), poly(ethylene oxide) (PEO), and polyurethane (PU) are the three most widely used SOEs materials,^[^ ^118^ ^]^ but still can be ignited once the temperature reaches a certain point.^[^ ^1^ ^]^ Their flammability can be effectively suppressed by physical blending and/or chemical bonding with polymeric flame retardants, such as TEP,^[^ ^119^ ^]^ decabromodiphenyl ethane (DBDPE) (see **Figure** **6**A),^[^ ^120^ ^]^ and hexa(4‐ethyl acrylate phenoxy) cyclophosphazene (HCP),^[^ ^121^ ^]^ and aluminum diethyl hypophasphate.^[^ ^122^ ^]^ In 2021, Long et al. reported a thermotolerant (no shrinkage at 200 °C) and fire‐proof gel polymer electrolyte in which the flame retardant, diethyl vinylphosphonate, was chemically bonded to the polymer matrix (see Figure 6B).^[^ ^123^ ^]^ This fire‐safe polymer electrolyte allowed for normal operation of LIBs at 150 °C or even exposure to butane flame. Likewise, Hu et al. also reported a similar flame‐resistant gel polymer electrolyte in which the polymerized form of tri(acryloyloxyethyl) phosphate (TAEP) and triethylene glycol dimethacrylate (TEGDMA) served as a flame retardant in the electrolyte (Figure 6C).^[^ ^124^ ^]^ In addition, the flame retardancy of polymer electrolytes can be improved by grafting flame‐retardant monomers into polymer chains.^[^ ^125^ ^]^ For instance, a modified polyurethane acrylate (PUA) electrolyte was prepared by grafting reactive flame‐retardant 9,10‐dihydro‐9‐oxa‐10‐phenanthrene‐10‐oxide (DOPO).^[^ ^126^ ^]^ Despite these advantages, these polymeric electrolytes suffer a low ionic conductivity which is normally 2 to 5 orders of magnitude lower than liquid electrolytes owing to the limited mobility of polymer chains and the rapid crystallization at room temperature.^[^ ^127^ ^]^ To improve the polymeric ionic conductivity, future research should focus on retaining the amorphous structure of the material at room temperature.

Non‐flammable polymeric ionic liquids have emerged as a promising fire‐safe option for SSEs. These materials utilize ionic liquid cations as the monomer, and the high concentration of salt in ionic liquids facilitates solid electrolytes with high ionic conductivity and transference number.^[^ ^129^ ^]^ In 2022, Chen et al. reported that solid electrolytes based on (poly(diallyldimethylammonium bis(fluorosulfonyl)imide (PDADMA FSI)) polymeric ionic liquid achieved fast alkali metal ion transport (1.0 × 10^−3^ S cm^−1^ at 80 °C) and a Na^+^ transference number of −0.57.^[^ ^129^ ^]^ This solvent‐free ionic liquid‐based solid electrolyte provides a new avenue for the development of high‐safety and high‐energy‐density AIBs.

Solid inorganic electrolytes (SIEs) commonly comprising ceramic materials exhibit superior thermal stability and ionic conductivity to the polymer‐based solid electrolytes, making them preferable for fire‐safe AIBs.^[^ ^130^ ^]^ However, Li dendrites often originate from the inhomogeneous surface contact between the hard SIEs and the Li metal anodes,^[^ ^131^ ^]^ and tend to grow along the grain boundaries in ceramics, leading to short circuits and mechanical failure of batteries.^[^ ^132^ ^]^ Also, the high‐energy‐density AIBs seek after dense thin film SIEs, but the ceramic materials are challenged by their poor processability.^[^ ^133^ ^]^

**Figure 7:**
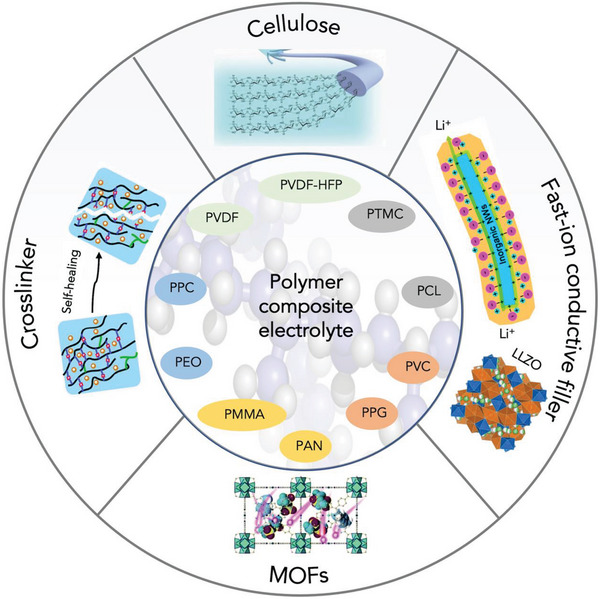
Various formulas to modify the polymer‐based SCEs for improved AIBs. Cellulose (Reproduced with permission,^[^ ^136b^ ^]^ Copyright 2018, Wiley‐VCH), MOFs as a host material for solid electrolytes (Reproduced with permission,^[^ ^139^ ^]^ Copyright 2017, Wiley‐VCH), fast‐ion conductive fillers Y_2_O_3_‐doped ZrO_2_ (Reproduced with permission,^[^ ^135a^ ^]^ Copyright 2016, American Chemical Society) and LLZO (Reproduced with permission,^[^ ^135b^ ^]^ Copyright 2018, American Chemical Society), physically cross‐linked network via ureidopyrimidinone (UPy) (Reproduced with permission,^[^ ^136a^ ^]^ Copyright 2018, The Royal Society of Chemistry).

The limitations of single‐phase SOEs or SIEs can be overcome by combining polymers and ceramics to make the most of both materials.^[^ ^134^ ^]^ For example, as summarized in **Figure** **7**, the ionic conductivity of polymer‐based electrolytes can be significantly enhanced by incorporating the fast ion conductor ceramic fillers (Li_7_La_3_Zr_2_O_12_ (LLZO) or Y_2_O_3_‐doped ZrO_2_) and metal–organic frameworks (MOFs) as host materials,^[^ ^135^ ^]^ and their mechanical properties can be improved by the addition of self‐healing cross‐linkers or cellulose.^[^ ^136^ ^]^ In contrast to SOEs the decomposition of which normally happens at 300 °C, most ceramic components in SCEs can keep in good shape at high temperatures and hence help sustain the integrity of electrolytes.^[^ ^137^ ^]^ On the other hand, the polymer in SCEs can protect the ceramic ionic conducting fillers (e.g., LLZO and sulfides, which are unstable in the air due to their high reactivity to CO_2_ and/or H_2_O), and endow the SCEs with intimate contact electrodes.^[^ ^138^ ^]^ Therefore, SCEs can provide excellent thermal and chemical stability for the fire‐safe AIBs with the formulations deliberately engineered.^[^ ^137^, ^138^ ^]^

Despite the nonflammability and thermal stability, SSEs are not necessarily safer than liquid electrolytes when battery failure occurs. Bates et al. reported that in the event of a short circuit, all‐solid‐state batteries with the LLZO electrolyte can even reach a significantly higher temperature than liquid LIBs, leading to fire through the flammable packaging and/or nearby materials.^[^ ^140^ ^]^ To ensure their safe use in industrial settings, it is equally important for all solid‐state batteries to undertake a series of quantitative thermal and fire safety tests, which will be discussed in the following section.

### 3.4 Separator Design

**Figure 8:**
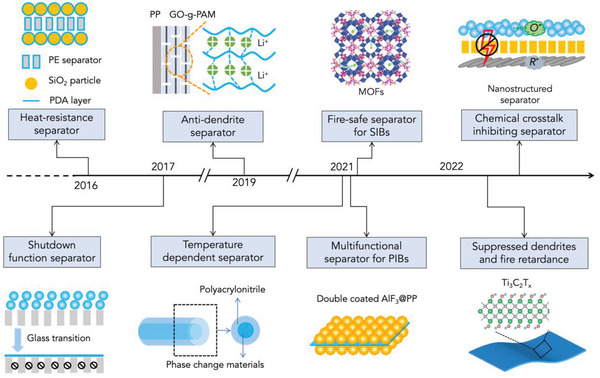
The recent progress in the development of safe separators for AIBs, including heat–resistance separator (Reproduced with permission.^[^ ^141^ ^]^ Copyright 2016, The Royal Society of Chemistry.), shutdown function separator (Reproduced with permission.^[^ ^2c^ ^]^ Copyright 2017, Wiley‐VCH.), anti–dendrite separator (Reproduced with permission.^[^ ^142^ ^]^ Copyright 2019, Springer Nature.), temperature dependent separator (Reproduced with permission.^[^ ^18^ ^]^ Copyright 2021, Wiley‐VCH.), multifunctional separator for PIBs (Reproduced with permission.^[^ ^7g^ ^]^ Copyright 2021, Wiley‐VCH.), fire–safe separator for SIBs (Reproduced with permission.^[^ ^143^ ^]^ Copyright 2021, Springer.), chemical crosstalk inhibiting separator (Reproduced with permission.^[^ ^144^ ^]^ Copyright 2021, Wiley‐VCH.), suppressed dendrites, and fire retardance separator (Reproduced with permission.^[^ ^145^ ^]^ Copyright 2022, Elsevier.).

The separators that are often of chemically and electrochemically inert materials are used to prevent the direct contact between anode and cathode in the battery, namely short circuits. Hence, their fire safety substantially determines the safety of the battery. For this reason, the past decade has seen great progress in the development of fire‐safe separators for AIBs (see **Figure** **8**).

The separators solely based on flammable PE can melt and shrink in the process of thermal runaway (>130°C), resulting in short circuits and fire/exploration.^[^ ^146^ ^]^ To address the issue of PE, one solution is to combine PE and polypropylene (PP) to form a PE‐PP bilayer or PP‐PE‐PP trilayer that can automatically shut down the battery under thermal abuse.^[^ ^8^, ^20^, ^147^ ^]^ The protection mechanism is that the PE film softens at 130 °C and collapses the pores in the separator, hence cutting off the transport of ions, while the PP layer with a higher melting point (165 °C) can maintain the integrity of the separator. The separator shut‐down should be triggered promptly before the onset of thermal runaway in batteries.^[^ ^146^, ^148^ ^]^ However, this type of composite separator cannot withstand a temperature greater than 165 °C, and hence it is necessary to improve the thermal resistance of separators, e.g., by adding polysulfone and SiO_2_ as additives to the polymer matrix.^[^ ^147^, ^149^ ^]^ The combination of bacterial cellulose and attapulgite in a composite material has emerged as a promising alternative to traditional PP separators.^[^ ^149g^ ^]^ Unlike PP, which shrinks severely at temperature above 150 °C, the bacterial cellulose‐attapulgite composite is capable of withstanding temperature up to 250 °C without any deformation. Additively, the attapulgite in the separator can play a crucial role during combustion by forming a dense carbon layer in the condensed phase. This layer protects the underlying material from external oxygen and helps to retard the flame. As a result, the bacterial cellulose‐attapulgite composite separator is a reliable and effective option for fire‐safe AIBs.

During the battery cycling, alkali ions shuttling between the electrodes pass through the porous channels in the separator back and forth,^[^ ^150^ ^]^ and the separator maintains as an electrical insulator unless they are breached by metal dendrites. To mitigate the dendrite‐related safety issues, the separator design is a promising method as it can manipulate the homogeneous flux of alkali ions and promote the deposition of a uniform dendrite‐free interface layer on the electrodes. The dendrite‐free separators are normally prepared by constructing an ordered porous structure in the separators,^[^ ^151^ ^]^ or combining them with lithiophilic materials (e.g., polar functional groups and MXene).^[^ ^142^, ^145^ ^]^ In addition, Han and co‐workers reported a dendrite‐resistant Mg(OH)_2_‐coated PP separator which converted Li metal dendrite into soluble Li^+^ ions by reacting with Mg(OH)_2_ nanoflakes once the two were in contact.^[^ ^152^ ^]^ The Mg(OH)_2_ coating can enhance the thermal stability and flame retardancy of the composite separator as well. Furthermore, Song et al. fabricated a nanoporous polyimide separator for LIBs,^[^ ^144^ ^]^ which is thermally stable and can effectively inhibit the chemical crosstalk of reactive gases, such as oxygen and hydrogen,^[^ ^153^ ^]^ between the anode and cathode (a cause of thermal runaway in batteries).^[^ ^8^, ^154^ ^]^

The conventional PP‐ and PE‐based separators are not suitable for SIBs and PIBs owing to their poor wettability to the electrolytes of high viscosity (e.g., PC) and the unwanted issues of dendrite growth.^[^ ^155^ ^]^ Recently, poly(vinylidene fluoride‐hexafluoropropylene) (PVDF‐HFP) based membranes have emerged as a promising fire‐safe separator matrix for SIBs, and the active fillers can be introduced to PVDF‐HFP to notably improve its ionic conductivity, mechanical property and thermal stability.^[^ ^156^ ^]^ Among these active fillers, the MOFs are of particular interest because of their good compatibility with the polymer matrices, and porous structures to enable fast ion transport.^[^ ^157^ ^]^ Recently, Feng et al. have incorporated the multifunctional UiO‐66 MOFs, which could uptake the electrolyte solution, selectively transport Na^+^ ions and provide flame retardancy, into the PVDF‐HFP membrane to form a fire‐safe, efficient separator for SIBs.^[^ ^158^ ^]^ To improve the electrochemical performance and safety of PIBs, Liu et al. also demonstrated a versatile PP separator coated with a reactive microscale AlF_3_ layer, which showed a thorough electrolyte wetting and an enhanced electrolyte uptake.^[^ ^7g^ ^]^ More importantly, the AlF_3_ coating also contributed to the formation of a stable uniform SEI layer on the K metal anodes, which helps reduce the dendrite growth and “dead metal” during the plating and stripping process.^[^ ^159^ ^]^

The above available separator design strategies can enhance their nonflammability and thermal stability, suppress the chemical crosstalk between electrodes, and modulate the alkali ion transport to solve dendrites concern. The synergism of these structural and chemical constructions in separator will be of great significance for the development of safe AIB system.

## 4 Thermal Management of AIBs

In addition to the above rational design of non‐flammable and fire‐extinguishing materials as the main battery components of fire‐safe AIBs, conventional safety devices, such as CID, PTC elements, and safety vents, are also commonly used to avoid short circuits, overcharging, and swelling of batteries.^[^ ^8^, ^160^ ^]^ Given the unpredictable operation environment for grid energy storage and electric vehicles, both reliable state monitoring and thermal management of batteries are also called for to maintain the optimal working temperature (typically between 25 and 40 °C),^[^ ^161^ ^]^ and to protect against the abuse situations and the ensuing thermal runaway.

### 4.1 Monitoring, Early Detection, and Prediction of Thermal Runaway

The current BMS typically consisting of multiple electronic units (e.g., sensors, actuators and collectors)^[^ ^161a^ ^]^ can monitor the status of each cell in the battery pack, such as voltage, current, temperature, state of charge (SOC), and state of health (SOH).^[^ ^8^, ^162^ ^]^ Thermal runaway early detection methods primarily rely on monitoring the temperature of battery cells to prevent them from reaching the onset of thermal runaway. Another approach involving the BMS detecting gas release as a sign of thermal runaway.^[^ ^8^, ^162^ ^]^ To address the issue of overcharging, Lyu et al. developed an on‐board dynamic impedance measuring device that can detect overcharge and prevent thermal runaway.^[^ ^163^ ^]^

However, the responsiveness of these early detection systems is limited by the surface‐mounted temperature sensors and voltage sensors because they cannot reflect the internal state of cells in real‐time.^[^ ^164^ ^]^ In the early 2010s, for the first time, temperature sensors for controlling LIB safety were demonstrated to shut down a battery short circuit 3 times earlier than the surface‐mounted sensor.^[^ ^165^ ^]^ In 2019, Li et al. incorporated an RTD into a 3D‐printed spacer that was directly attached to the cathode in LIBs.^[^ ^16^ ^]^ Compared to the external RTD, this embedded RTD battery design showed an average 5.8 °C higher internal temperature of LIBs and 10 times faster temperature detection speed, thus offering a more precise tool for monitoring the thermal runaway of batteries. They further developed a reliable temperature prediction model which could diagnose the short circuit by correlating the external RTD measurement to the internal RTD measurement.

Apart from the temperature detection, the gas signals, related to volatile organic compounds in the event of electrolyte leakages, are another key indicator for predicting battery failures and fires.^[^ ^166^ ^]^ Gas sensors, therefore, are very useful in the early detection of thermal runaway in batteries.^[^ ^167^ ^]^ The qualitative and quantitative gas‐phase characterizations have revealed that the thermal runaway of AIBs generally produces H_2_, CH_4_, C_2_H_4_, CO and CO_2_, and the release rates of these gases and their concentrations will sharply increase when the metallic casing cracks.^[^ ^153^, ^166^, ^168^ ^]^ With the representative chemical database, the specific gas sensors can be readily integrated into one system to monitor the gaseous atmosphere in batteries, thus allowing for a timely early warning of thermal runaway.^[^ ^169^ ^]^

Various models have been proposed for predicting battery thermal runaway based on kinetics studies.^[^ ^8^, ^161^, ^170^ ^]^ Ren et al. established a battery thermal runaway model by coupling all the exothermic reactions of the battery during thermal runaway, and this model's predictions were consistent with the thermal runaway test results of a 24 Ah LIB, indicating its reliability.^[^ ^171^ ^]^ Melcher et al. developed an electrochemical‐thermal model that can predict the onset temperature of thermal runaway, which is very close to the temperature at which the SEI layer starts to decompose.^[^ ^172^ ^]^ Feng et al. built an electrochemical‐thermal model that can predict battery capacity degradation, internal short circuit, and chemical reactions between battery components under thermal runaway.^[^ ^173^ ^]^ Ostanek et al. developed a thermal runaway model by coupling the thermal and gas venting model,^[^ ^174^ ^]^ which can simulate the heat generation and gas expulsion during battery heating. However, for these thermal runaway prediction models to be widely applied, more systematic monitoring and analysis of the electrochemical‐thermal coupling characteristics of batteries during thermal runaway are necessary to establish the models.

### 4.2 Battery Thermal Management

Thermal management systems are used to ensure the entire battery system works in a safe, mild temperature range by dissipating the heat generated from batteries during operation.^[^ ^162^, ^175^ ^]^ Traditional thermal management systems often take advantage of air cooling, liquid cooling, cold plates and/or cooling fins to promote the heat transfer from the battery to the exterior environment.^[^ ^176^ ^]^ However, these cooling units often significantly increase the weight, cost and volume of batteries and lead to more parasitic energy loss, due to the inclusion of pumps, valves, and heat exchangers in the thermal management system.

Alternatively, passive or semi‐passive thermal management supported by PCMs does not need these auxiliary cooling components, thus holding great promise for heat dissipation in batteries.^[^ ^177^ ^]^ In these systems, PCMs can absorb or release latent heat at the phase change temperature at which melting or solidification occurs. Because of this unique merit, the PCM‐based thermal management exhibits an ability to effectively buffer and store the thermal energy from batteries in operation while keeping batteries warm in a cold environment.^[^ ^177d^ ^]^ Therefore, this passive thermal management system can boost both battery safety and battery performance to some extent. The main drawback of PCMs, such as paraffin, is the low thermal conductivity, which goes against heat dissipation and absorption.^[^ ^177^, ^178^ ^]^ One promising solution to this drawback is the addition of expanded graphite, graphene or metals to PCMs to improve their thermal conductivity.^[^ ^179^ ^]^ Compared to paraffin, more cost‐effective sodium thiosulfate pentahydrates (STP) are advantageous in higher thermal conductivity and an appropriate melting point (47 °C) within desired battery operating temperature range.^[^ ^180^ ^]^ The application of PCMs in battery packs, especially for electric vehicles, is still in its initial stage. Even though their thermal behavior allows for effective battery temperature management, most paraffin‐based PCMs are flammable, calling for more efforts to minimize their flammability.

## 5 Fire Safety Evaluation

The fire safety evaluation of AIBs is equally important to their fire‐safety design. It can be generally divided into four categories: a) the thermal stability characterization of battery materials and thermal runaway characteristics of batteries under conditions of thermal abuse,^[^ ^12^, ^26^, ^181^ ^]^ b) flammability tests of battery materials,^[^ ^191^ ^]^ c) abuse tests under thermal, mechanical and electrical abuse conditions, and d) appraisal of fire‐induced hazards of batteries.

### 5.1 Thermal Behavior Characterization

Thermal behavior characterization involves the evaluation of both the thermal stability of battery materials and the thermal runaway of batteries under thermal abuse conditions. From a point of view of practical applications, it is vital to develop effective discriminative methods to characterize the thermal behaviors of individual battery components and the whole batteries for the design of fire‐safe AIBs.^[^ ^182^ ^]^

**Figure 9:**
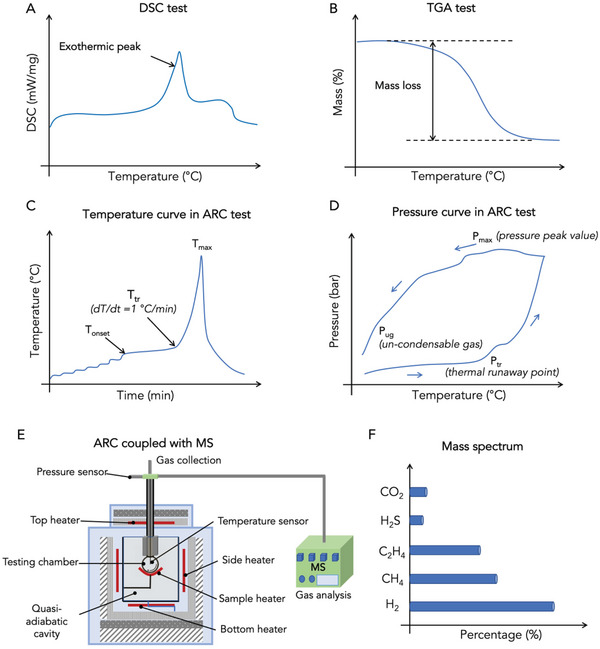
Thermal behavior characterization techniques. A) DSC, B) TGA, C) temperature, and D) pressure profiles under heat‐wait‐search test in ARC, E) schematic illustration of ARC‐MS system, and F) mass spectrum determined by MS after the thermal runaway. Reproduced with permission,^[^ ^26c^ ^]^ Copyright 2022, Elsevier.

The exothermic behaviors of electrode materials, electrolytes and the complete battery can be characterized by differential scanning calorimetry (DSC) or C80 calorimetry.^[^ ^182^, ^183^ ^]^ For instance, Duh et al. used DSC to study the individualized reactivity of cathode materials with EC in LIBs, and the direct observation of the exothermic reaction reveals the possible thermal runaway routes in batteries (**Figure** **9**A).^[^ ^182^ ^]^ Because of the higher sensitivity than DSC in the lower temperature range (from room temperature to 300 °C), the C80 calorimeter is also employed to detect the exothermic reaction of electrolytes with cathodes of LIBs.^[^ ^183^, ^184^ ^]^

In terms of the thermal stability of the complete battery, Inoue and Mukai developed an all‐inclusive microcell that possessed all battery components and worked as a battery by itself. The all‐inclusive microcell was tested by DSC to determine the total heat generation (ΔH), which is used to evaluate the battery safety by comparing it with that of conventional LIBs.^[^ ^185^ ^]^ Although both DSC and C80 calorimetry can be effective in measuring the temperature and heat absorption/release of substances, they cannot identify the decomposition reactions in batteries. To this end, the quantitative thermogravimetric analysis (TGA) coupled with the mass spectrometry (MS) method is introduced to reveal the decomposition mechanism of battery materials by measuring the mass change and the rate of mass change of substances under heating (Figure 9B) as well as gas evolution of the substances.^[^ ^186^ ^]^ In addition, Fourier transform infrared spectrometer (FTIR) can be combined with the C80 calorimetry to shed light on the exothermic reaction mechanism.^[^ ^183d^ ^]^

The heat generated from the exothermic reactions between electrodes and electrolytes generally follows an exponential function, while heat dissipation presents a linear one.^[^ ^7f^ ^]^ When the heat generated from the reactions outweighs the heat dissipation capability of the battery, the exothermic processes will proceed under adiabatic‐like conditions and the temperature of the battery will increase exponentially.^[^ ^187^ ^]^ Therefore, it is essential to examine the thermal behaviors of batteries and their components under adiabatic conditions. To this end, calorimetric methodologies, typically vent sizing package 2 (VSP2) and ARC, have been extensively used for the measurement of temperature‐pressure‐time profiles.^[^ ^29^, ^188^ ^]^

A vent sizing package 2 (VSP2) adiabatic calorimeter is largely used to study the thermal deterioration of a whole battery, such as prismatic and 18650 LIBs.^[^ ^29^, ^188^, ^189^ ^]^ Similarly, ARC tests are conducted on either battery components or complete batteries in a heat‐wait‐search mode to measure thermal runaway characteristics (Figure 9C), including the onset temperature for self‐heating (*T*_onset_), self‐heating rate (dT/dt), thermal runaway point (*T*_tr_, which is defined to be the point when self‐heating rate is 1 °C min^−1^), and the maximum temperature (*T*_max_).^[^ ^190^ ^]^ The thermal runaway of a battery is reported to be affected by the inner pressure or cross‐talk of heat‐induced gases.^[^ ^26c^ ^]^ The different thermal runaway mechanisms in AIBs can be elucidated using the pressure‐temperature profile under the heat‐wait‐search test as well as the detection of non‐condensable gas species by MS after the heat‐wait‐search test in ARC (Figure 9D–F).^[^ ^26c^ ^]^

### 5.2 Flammability Characterization

The flammability characteristics of liquid‐state electrolytes,^[^ ^191^ ^]^ including flash point, auto‐ignition temperature, limiting oxygen index (LOI) and self‐extinguishing time (SET) are normally used for the evaluation of the flammability features.^[^ ^191b^ ^]^ The flash point test relies on the ignition of the vapor‐air space formed above the heated liquid substance. The lowest temperature at which ignition occurs is regarded as the flash point.^[^ ^192^ ^]^ Without the application of an additional external ignition source, an increased temperature can also provide sufficient heat energy to ignite the substance, and the temperature to ignition is defined as the auto‐ignition temperature.^[^ ^192^ ^]^ The LOI testing (GB/T 16581‐1996) is carried out by igniting the electrolyte in a burning cup and recording the time taken for the flame to extinguish under an O_2_/N_2_ mixture atmosphere. In LOI testing, the O_2_ concentration will be tuned until the time to extinguish reaches 60 s, and this oxygen percentage is recorded as the LOI value of a specific material.^[^ ^88d^ ^]^ The SET is commonly used to evaluate the flame‐retardant performance of electrolytes. The test is performed by igniting a cotton ball‐wick (diameter: 0.3–0.5 cm) which absorbs electrolyte (0.05–0.10 g) and recording the time taken for the flame to extinguish. The SET value is calculated by normalizing the burning time against the electrolyte mass, and the SET testing results make a better distinction for non‐flammable electrolytes as compared to the other above‐mentioned tests.^[^ ^90^ ^]^

As for the flammability testing of solid‐state materials for AIBs, such as separators,^[^ ^193^ ^]^ SSEs,^[^ ^194^ ^]^ and binders in electrodes,^[^ ^195^ ^]^ direct flame exposure is generally applied. During the testing, the burning time normalized by material mass is used for assessing the flame‐retardant property of the material.^[^ ^195^ ^]^ It should be noted that the flammability of the battery vent gas should be carefully considered because the fire risk of AIBs can be attributed to the flammable battery vent gas, e.g., such as CO, H_2_, CH_4_, C_2_H_4_, C_2_H_6_.^[^ ^196^ ^]^ The flammability characteristics of the battery vent gases include the flammability limit, flammability hazard index, fire safety threshold, and diluent ratio threshold, all of which have been detailly studied by Wang et al. for safer LIBs under an external heating abuse.^[^ ^196a^ ^]^ This meaningful work verified a practical measure to change the battery vent gas from flammable to non‐flammable by using CO_2_ dilution, which is of high value for preventing battery thermal runaway.

### 5.3 Abuse Testing

The abuse testing of AIBs by exposing them to abnormal conditions can provide a deep insight into the probability and severity of a thermal runaway event. A variety of abuse tests have been applied to AIB products by mimicking thermal, mechanical, and electrical abuse scenarios, as summarized in **Table** **1**.^[^ ^197^ ^]^

**Table 1:** Reported abuse testing of AIBs under varied abuse conditions

Abuses type	Test method	Key testing parameters	Test results	Ref.
Thermal	Thermal shock	75–115 °C, 30 min; and −40 °C, 30 min	The failure (venting, battery enclosure rupture, internal resistance, fire, or explosion) of the cell.	[198]
	Temperature aging	low temperature (0 °C) high temperature (55 or 85 °C).	The influence of aging on the batteries.	[199]
	Thermal ramp test	The test is performed in an oven until the cell is either shorted or exploded. the heating rate of 3 °C min^−1^ room temperature to 180 °C or higher	Record time and temperature until the cell failed.	[200]
	Heating abuse	thermal runaway temperature	Thermal runaway process, the temperature and voltage change of the cell under external heating abuse	[201]
Mechanical	Altitude simulation	The cell is placed in a container that produces an atmospheric condition. the pressure of 11.6 kPa or less simulating an altitude of 10 000 m or more over the sea level.	The failure (leakage, vent, and open circuit potential) of the cell.	[202]
	Vibration	United States advanced battery consortium recommends random and sine harmonic vibration. European regulation ECE R100.2 involves swept sine vibration in the Z direction.	The open circuit potential, temperature, gaseous release, morphology of electrodes, and battery performance.	[203]
	Shock	force acceleration shock duration	The robustness of the cell in the situation of sudden acceleration and/or deceleration of its powering devices.	[203, 204]
	Free fall	a height of 10 cm 3 times.	Leakage and voltage drop, the temperature increase of the cell after free fall.	[205]
	Nail insertion	nail with a diameter of 2.5 mm penetration down to the half depth of the cell	Battery short‐circuits, ignition, or explosion.	[205]
	Crush test	crushing force	Battery short‐circuits, and/or temperature increases.	[206]
Electrical	External terminal short	a cooper wire of which the resistance is adjustable to connect the electrodes	Evaluate the temperature increase, leakage, swelling, fire, and even explosion.	[207]
	Overcharge	the cell without PTC a compliance discharge voltage at which the cell reached more than 100% of its discharged capacity.	Evaluate the battery swelling, temperature increase, short‐circuiting, fire, or explosion.	[7, 208]
	Over discharge	a voltage below the working potential window even with negative polarity normal cycling test after abuse	Internal battery resistance and battery performance degradation.	[209]

**Figure 10:**
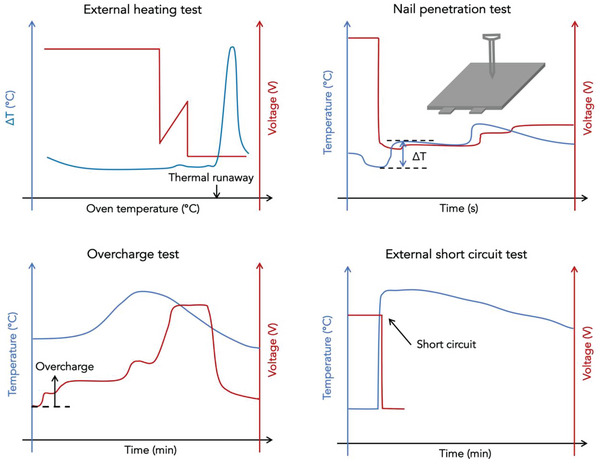
Thermal (external heating), mechanical (nail penetration), and electrical (overcharge and external short circuit) abuse trials on AIBs.

Battery temperature and voltage are two key parameters for monitoring the battery status under abusive scenarios. **Figure** **10** shows the temperature and voltage profiles of the battery in four typical abuse tests. In the external heating abuse test, the Y‐axis (Δ*T*) indicates the temperature difference between the oven temperature (which is heated up to *T*_tr_) and the battery temperature, and the maximum Δ*T* can be used to evaluate the thermal safety of batteries.^[^ ^210^ ^]^ The detected voltage drop in the thermal runaway process indicates the battery short‐circuit which is likely ascribed to the melting and rupture of separators.^[^ ^146^, ^211^ ^]^ Nail penetration, overcharge, and external short circuit abuse tests are operated on different battery systems (such as Li‐S batteries and LIBs) to evaluate their fire safety and aid in the analysis of their thermal runaway mechanisms.^[^ ^26c^ ^]^ Meanwhile, they can also determine the safety of battery components, including separator,^[^ ^200^ ^]^ electrolyte,^[^ ^202a^ ^]^ electrodes,^[^ ^203a^ ^]^ and packaging materials.^[^ ^201^ ^]^

### 5.4 Appraisal of Fire‐Induced Hazard of Battery

**Figure 11:**
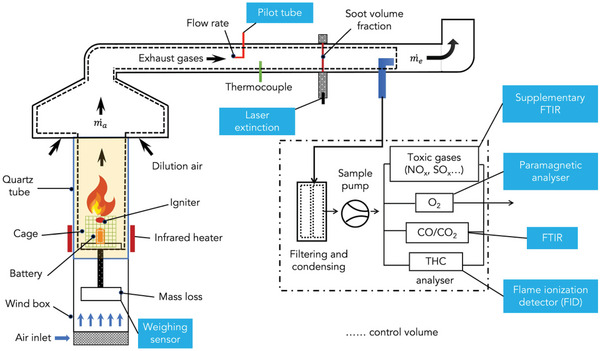
Schematic of the fire propagation apparatus (Tewarson calorimeter). Reproduced with permission,^[^ ^181c^ ^]^ Copyright 2012, The Royal Society of Chemistry.

The burning behavior, mass loss, heat release rate (HRR), and total heat released (THR) of battery packs are critical parameters for assessing the flammability of batteries in a real fire scenario.^[^ ^7e^ ^]^ Fire propagation apparatus, also known as Tewarson (Cone) calorimetry in the EU, is also used for the combustion testing of pouch cells (**Figure** **11**). Ribièrethe et al. investigated the fire‐induced hazards of commercial LIB by fire calorimetry, which allows for the online analysis of mass loss as well as combustion gas production. The HRR can be calculated based on O_2_ consumption corrected for CO and soot production, and the peak HRR value tends to be higher with increasing SOC.^[^ ^181c^ ^]^ Considering the high toxic halogen halides in the toxic emissions, from the fire safety point of view, current research has been focused on replacing the fluorine‐containing binders with low‐toxic carboxymethyl cellulose,^[^ ^212^ ^]^ and using fluorine‐free electrolytes.^[^ ^181^, ^213^ ^]^ To make this section more intelligible, **Table** **2** summarizes some representative fire safety characterization methods and corresponding characterized properties of AIBs.

**Table 2:** A summary of up‐to‐date applied fire safety characterization for AIBs

Characterization method	Component	Properties characterized	Battery system (anode||cathode)	Ref.
DSC	Graphite anode	Thermal behavior of different charged states graphite anode	Li||Graphite	[181a]
	Graphite anode	Thermal event of anode with different lithiation states, binders (PVDF), and electrolytes	Graphite||Li_0.3_NiO_2_	[214]
	Li_0.3_NiO_2_ cathode	Thermal stability of delithiated cathode, and substituted nickel‐based cathode materials		
	LiPF_6_ in PC/EC/DMC electrolyte	The decomposition behavior of electrolytes under heating treatment		
	Separator PP and/or PE	Thermal contribution of separator in complete cell		
DSC and ARC	Complete cell	The heat source in the thermal runaway process and the thermal runaway mechanisms	Li||S LIBs	[26, 215]
DSC‐TGA	Li_2_MnSiO_4_ cathode	Thermal stability of fresh or cycled Li_2_MnSiO_4_ in LiPF_6_‐based electrolyte	Li||Li_2_MnSiO_4_	[216]
	NaCrO_2_ cathode	The heat generation and release of O_2_ during heating	Na||NaCrO_2_	[217]
	Hard carbon anode with electrolytes	The thermal behaviors of mixtures of sodiated/lithiated hard carbon anode and EC‐EMC‐based or PC‐based electrolytes.	Na||Hard carbon Li||Hard carbon	[218]
	Li_2_MnSiO_4_ cathode	The thermal stability of fresh and cycled Li_2_MnSiO_4_ cathode as well as its charged and discharged products in LiPF_6_‐based electrolyte	Li||Li_2_MnSiO_4_	[216]
TGA	NaPF_6_/BMITFSI electrolyte	thermal stability of ionic liquid electrolyte	Na||Na_3_V_2_(PO_4_)_3_	[219]
	C_8_H_6_O_4_ cathode	thermal stability of cathode material	K||C_8_H_6_O_4_	[220]
SET	Polyimide‐based current collector	The flame retardancy of polyimide‐based current collector/graphite anode soaked with electrolyte	Graphite||LiCoO_2_	[221]
	NaPF_6_/BMITFSI electrolyte	non‐flammability of ionic liquid electrolyte	Na||Na_3_V_2_(PO_4_)_3_	[219]
LOI	Polyurethane solid electrolyte	flame retardancy of polyurethane electrolyte	Li||LiFePO_4_	[181b]
ARC	Complete cell	The thermal runaway characteristics (self‐heating onset temperature, thermal runaway temperature and the maximum temperature) of a full cell	Hard carbon||NaNi_1/3_Fe_1/3_Mn_1/3_O_2_	[222]
MS	Heat‐induced gas liberations of battery components	Non‐condensable gas species after ARC test	Li||S	[26c]
VSP2 adiabatic tests	LiCoO_2_ and Li(Ni_1/3_Co_1.3_Mn_1.3_)O_2_ cathodes, and complete cell	Characteristics of self‐reactive rating of cathodes and complete batteries (initial exothermic temperature (*T*_0_), self‐heating rate (dT/dt), pressure rise rate (dP/dt), maximum temperature (*T*_max_) and pressure (*P*_max_))	Graphite||LiCoO_2_ Graphite||Li(Ni_1/3_Co_1.3_Mn_1.3_)O_2_	[188c]
C80 micro‐calorimeter	LiPF_6_ in EC/DEC electrolyte, dissembled complete cell	The onset temperature and the temperature of exothermic peaks	Graphite||LiCoO_2_	[183e]
Cone calorimeter	Complete cell	The temperature, HRR, gas release, mass loss, and residue during the thermal runaway process	Graphite||LiNi_x_Co_y_Mn_1‐x‐y_O_2_	[223]
Short‐circuit	Complete cell	The voltage and temperature evolutions	Pyrolyzed anthracite||Na_0.9_[Cu_0.22_ Fe_0.30_Mn_0.48_]O_2_	[224]
Nail penetration				
Overcharge				

^a)^ DMC = dimethyl carbonate;

^b)^ BMITFSI = 1‐butyl‐3‐methylimidazolium bis (trifluoromethanesulfonyl) imide.

## 6 Conclusion and Outlook

### 6.1 Conclusions

In summary, great advances have been made in creating fire‐safe AIBs so far. The ultimate solution to the fire issue of AIBs is the combination of intrinsic fire‐safe battery materials and efficient external thermal management. The design of fire‐safe anode materials largely relies on the application of safer non‐alkali metal anodes, such as graphite and silicon anodes for LIBs, hard carbon for SIBs, graphite and metal phosphide for PIBs. In terms of the design of fire‐safe cathode materials, existing strategies mainly focus on suppressing the decomposition of active materials and the release of oxygen at elevated temperatures. To enhance the fire safety of both anode and cathode, thermally responsive polymers and/or fire‐retardant additives can be used to cut off the electric connection under high‐temperature abuse and/or impart flame retardancy to the electrodes, respectively.

Liquid organic electrolytes can be engineered to be non‐flammable and highly ionic conductive which helps impede the growth of metal dendrite. Meanwhile, intrinsically fire‐safe aqueous electrolytes are also explored to extend their narrow electrochemical stability window. Fire‐safe SSEs, including SOEs, SIEs and SCEs, are leak‐proof, separator‐free and non‐volatile, which are intensively investigated toward low flammability, high thermal stability, and robust mechanical properties. SCEs are more attractive because of the synergy between ceramic ionic conducting fillers and polymer matrices resulting in improved ionic conductivity and lower interfacial impedance with electrodes. With respect to separators used in liquid‐state electrolytes, multifunctional separators with excellent electrolyte uptake property, shut‐down features, high thermal stability, flame retarding, and alkali‐ion transport modulating functions have been developed for underpinning the fire‐safe battery design.

Thermal management of AIBs can manage the thermal behavior of the battery by controlling voltage, current, pressure, and temperature to maintain the safe operation of batteries. Active protection is a prerequisite to eliminating the risk timely before it turns uncontrollable. Early warning and monitoring by safety devices mounted in batteries can diagnose the short circuit and thermal runaway and prevent a fire hazard. PCMs, which can store and release thermal energy through the phase change process, are applied to AIB packs to maintain the battery temperature in a normal operating range without using any external power.

A comprehensive fire safety evaluation system is also essential to assess the safety of battery components and the complete battery by better casting light on the thermal runaway process of batteries. A variety of advanced thermal property analysis techniques (e.g., DSC, TGA, ARC, and cone calorimetry) and flammability tests (e.g., SET, and LOI) can characterize the thermal stability and flammability of battery materials and the complete cells in a qualitative and/or quantitative manner. The abuse testing conducted on batteries can characterize their limitations facing external abuses as well as assist in the investigation and evaluation of their thermal runaway process in applications.

### 6.2 Outlook

**Figure 12:**
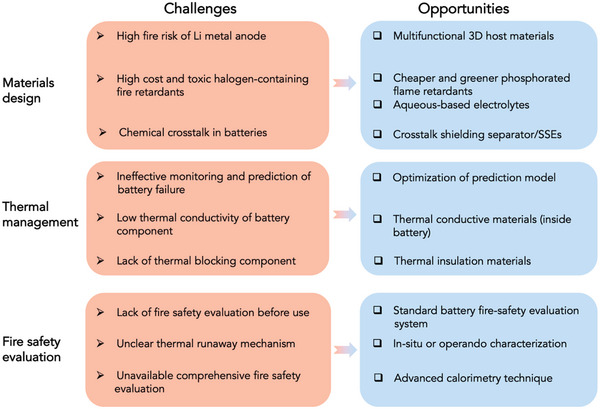
Current challenges and the opportunities for next‐generation fire‐safe AIBs.

Although the last years have witnessed great advancements in creating fire‐safe AIBs, some key challenges remain to be addressed, as illustrated in **Figure** **12**. From the aspect of battery materials design, Even though the dendrites growth issue in graphite anode in LIB under high rate could be solved, graphite anodes in LIBs are bottlenecked by limited energy density. Li metal anode with extremely high energy density is attractive for making smaller and lighter rechargeable batteries. Fire‐retarding and thermal dissipation host scaffolds for alkali metals which can also suppress the dendrite growth can be developed to enhance the fire safety of anodes. As for organic‐based electrolytes, halogen‐functionalized fire‐retardant additives are not cost‐effective to fulfill industry requirements, while greener and cheaper phosphorated flame retardants are preferred. Meanwhile, with the development of aqueous‐based electrolytes with a wider working potential window for LIBs, more attention should be focused on the exploration of aqueous‐based electrolytes for SIBs and PIBs due to their high safety and low cost. To inhibit chemical crosstalk‐induced thermal runaway in batteries, chemical modification of nano porous separators or SSEs could be exploited, for instance, by incorporating transition metal ion adsorption materials (e.g., graphene or mXene) in polymer‐based SSEs, the chemical crosstalk can be better blocked.

The safety devices mounted inside the cell to monitor and predict the battery failure,^[^ ^225^ ^]^ are superior to the surface‐mounted ones in terms of delivering a quicker response, which will be further improved in terms of the fabrication cost and large‐scale application. Embedded thermal sensors inside batteries were used for detecting and monitoring the internal health conditions and the safety limits of batteries.^[^ ^226^ ^]^ The temperature, as well as the electrochemical performance data during their early service life, are valuable inputs for constructing a more accurate prediction model using machine learning, which can be used to predict the failure time and failure impact factors.^[^ ^227^ ^]^ Machine learning as an efficient tool for predicting battery life will be more extensively applied in the safety management of AIBs.

The conventional thermal management materials are normally applied outside the battery to dissipate heat from the surface of the battery, but they can cause an unwanted increase in temperature and thermal gradient inside the battery. In comparison, internal cooling management is preferable to improve safety and durability,^[^ ^228^ ^]^ of which the investigation is still in its early stage, so more efforts should be put into this direction in future research. Meanwhile, the thermal conducting capability of the battery components should be improved by using highly thermally conductive materials in electrolytes and electrodes, such as BN, graphene, and carbon nanotube. In addition, thermal insulation materials used between modules of the battery pack are of high commercial and academic interest, because they can efficiently block the heat transfer between the modules and thus stop the fire from propagating.^[^ ^229^ ^]^ The potential for fire hazards during battery transportation, storage, and recycling operations can be significantly higher than in end‐use applications, particularly due to condensation caused by temperature changes. For example, battery containers being moved from a cold place to a heated warehouse can increase the risk of condensation. Packaging instructions require a safety vent to prevent explosions and measures to prevent external short circuits. Additionally, the United Nations recommends that batteries undergo a series of safety tests prior to transport.^[^ ^230^ ^]^ Research into the transportation and storage of end‐of‐life batteries is also an essential area to consider beyond the serving period of AIBs. According to a report by Slattery et al. in 2021, there is a need for policy or future research in the collection and transportation of end‐of‐life LIBs.^[^ ^227^ ^]^ Accurately testing the state‐of‐health of recycled batteries and classifying them will facilitate better safety management.

In‐operando high‐speed characterization techniques are being more extensively applied to reveal the thermal runaway mechanisms. For instance, transmission electron microscopy,^[^ ^231^ ^]^ X‐ray absorption and mass spectroscopy,^[^ ^232^ ^]^ synchrotron X‐ray diffraction^[^ ^233^ ^]^ are applied to determine the materials’ physical structure and chemical composition changes in the operating batteries. In‐operando X‐ray inspection systems combined with thermal imaging,^[^ ^35^, ^234^ ^]^ Raman,^[^ ^235^ ^]^ and FTIR are also deployed to examine the thermal impact on chemical composition, structural degradation, and gas emission during initiation and propagation of thermal runaway in complete cells.^[^ ^26^, ^236^ ^]^ These advanced in‐operando characterization methods will continually assist in designing safer battery materials.

Despite some encouraging advancements, there remains a long way to go in the materials design, thermal management, and fire safety evaluation for the creation of fire‐safe AIBs. Particularly, in the future, more accurate methods need to be developed for the characterization and evaluation of fire safety of batteries during deployment in real‐life scenarios, and for better assessment and understanding of battery materials and battery chemistry. We envision that more systematic and comprehensive fire safety evaluation and more efficient characterization techniques hold the promise of leapfrog advances for next‐generation fire‐safe AIBs.

## Conflict of Interest

The authors declare no conflict of interest.
